# Best practices for time-resolved serial synchrotron crystallography

**DOI:** 10.1107/S2059798321011621

**Published:** 2022-01-01

**Authors:** Eike C. Schulz, Briony A. Yorke, Arwen R. Pearson, Pedram Mehrabi

**Affiliations:** a Institute for Nanostructure and Solid State Physics, Universität Hamburg, HARBOR, Luruper Chaussee 149, 22761 Hamburg, Germany; b School of Chemistry and Bioscience, University of Bradford, Bradford BD7 1DP, United Kingdom; c Hamburg Centre for Ultrafast Imaging, Universität Hamburg, HARBOR, Luruper Chaussee 149, 22761 Hamburg, Germany

**Keywords:** time-resolved crystallography, serial crystallography, SSX, structural enzymology, mechanistic structural biology

## Abstract

With recent developments in X-ray sources, instrumentation and data-analysis tools, time-resolved crystallographic experiments, which were originally the preserve of a few expert groups, are becoming simpler and can be carried out at more radiation sources, and are thus increasingly accessible to a growing user base. However, these experiments are just that: discrete experiments, not just ‘data collections’. As such, careful planning and consideration of potential pitfalls is required to enable a successful experiment. Here, some of the key factors that should be considered during the planning and execution of a time-resolved structural study are outlined, with a particular focus on synchrotron-based experiments.

## Abstract 2

The key factors that should be considered during the planning and execution of a time-resolved crystallographic experiment are discussed, with a focus on time-resolved serial synchrotron crystallography.

## 1. Introduction

Macromolecular X-ray crystallography has become near-ubiquitous at synchrotrons, which often house multiple heavily automated beamlines dedicated to macromolecular crystallo­graphy, permitting routine in-house and even remote data collection without extensive user training. This success has been possible as a result of the increasing levels of automation in the field and the development of standardized workflows. These have resulted in an explosion in the number of protein structures deposited annually into the PDB and have enabled the emergence of related fields such as structural genomics and structure-based drug discovery. Very recently, this wealth of protein structure information has allowed machine-learning approaches to essentially solve the challenge of protein structure prediction from primary-sequence data (Tunyasuvunakool *et al.*, 2021 ▸; Baek *et al.*, 2021 ▸). With the recent release of the AlphaFold protein structure database (https://alphafold.ebi.ac.uk), hundreds of thousands of (predicted) protein structure models have been made available covering the majority of published protein sequences (Tunyasuvunakool *et al.*, 2021 ▸), and in time this is likely to increase to millions. This sudden abundance of structural information has highlighted that the main incentive for macromolecular structure determination is not the structure *per se*, but rather the increased understanding of the underlying biological function that the structure provides. We thus expect that mechanistic studies, and in particular those that focus on macromolecular dynamics, will see a resurgence in the coming years.

Combining conventional structural biology approaches with other biochemical or biophysical experiments has allowed the unravelling of biochemical mechanisms of proteins, for example via mutation studies, substrate analogues or freeze-trapping techniques (Moffat, 2001 ▸). However, while these classical X-ray crystallographic approaches have been extremely powerful in obtaining insight into equilibrium-state structures, structures obtained from mutants, or with substrate analogues or inhibitors, have the potential to reflect artefacts that only exist in the mutant or particular ligand complex, particularly when trying to probe a reaction mechanism by determining the structure of reaction intermediate-like states. These can go unnoticed when follow-up studies with other techniques or on wild-type proteins are not carried out (Moffat, 2001 ▸). In addition, while the stabilization of certain reaction intermediate states can be achieved with substantial modification of the target protein, many intermediate states are either impractical or simply impossible to obtain by static and trapping methods.

For a comprehensive insight into macromolecular function it is imperative to combine structural information with insight into the time-dependent changes, in other words the dynamics, of the protein of interest. This information can not only reveal metastable intermediates that are difficult to trap, but can also reveal hitherto invisible features of protein function, providing novel insights into catalysis, allostery, half-of-the-sites reactivity, oxidation states, side-chain motions, isomerization reactions and molecular breathing, as well as into molecular binding events (Weinert *et al.*, 2019 ▸; Nango *et al.*, 2016 ▸; Nogly *et al.*, 2018 ▸; Nass *et al.*, 2020 ▸; Arnlund *et al.*, 2014 ▸; Coquelle *et al.*, 2018 ▸; Barends *et al.*, 2015 ▸; Kupitz *et al.*, 2014 ▸; Tenboer *et al.*, 2014 ▸; Stagno *et al.*, 2017 ▸; Pande *et al.*, 2016 ▸; Mehrabi, Schulz, Dsouza *et al.*, 2019 ▸; Kern *et al.*, 2013 ▸).

With the introduction of time-resolved crystallography nearly 30 years ago, pioneers in the field harnessed polychromatic X-rays at synchrotron sources to obtain Laue diffraction patterns, ultimately pushing the time resolutions for time-resolved protein crystallography into the picosecond regime (Ren & Moffat, 1994 ▸; Ren *et al.*, 1999 ▸). However, it was not until the advent of X-ray free-electron lasers (XFELs) and the widespread adoption of serial crystallographic techniques that a wider interest in time-resolved macromolecular crystallography emerged (Levantino *et al.*, 2015 ▸; Pearson & Mehrabi, 2020 ▸; Orville, 2020 ▸).

Soon after its exploitation at XFELs, serial crystallography was rapidly translated to microfocus beamlines at synchrotrons and serial synchrotron crystallography (SSX) was born (Boutet *et al.*, 2012 ▸; Chapman *et al.*, 2011 ▸; Redecke *et al.*, 2013 ▸; Barends *et al.*, 2014 ▸; Arnlund *et al.*, 2014 ▸; Gati *et al.*, 2014 ▸; Roedig *et al.*, 2016 ▸; Botha *et al.*, 2015 ▸; Stellato *et al.*, 2014 ▸). Serial crystallography has a number of advantages for time-resolved diffraction data collection; for example, it avoids the need for reversible systems (Chapman, 2019 ▸; Weinert *et al.*, 2017 ▸; Schulz *et al.*, 2018 ▸; Mehrabi, Schulz, Agthe *et al.*, 2019 ▸; Mehrabi, Schulz, Dsouza *et al.*, 2019 ▸; Nogly *et al.*, 2015 ▸).

**Figure 1:**
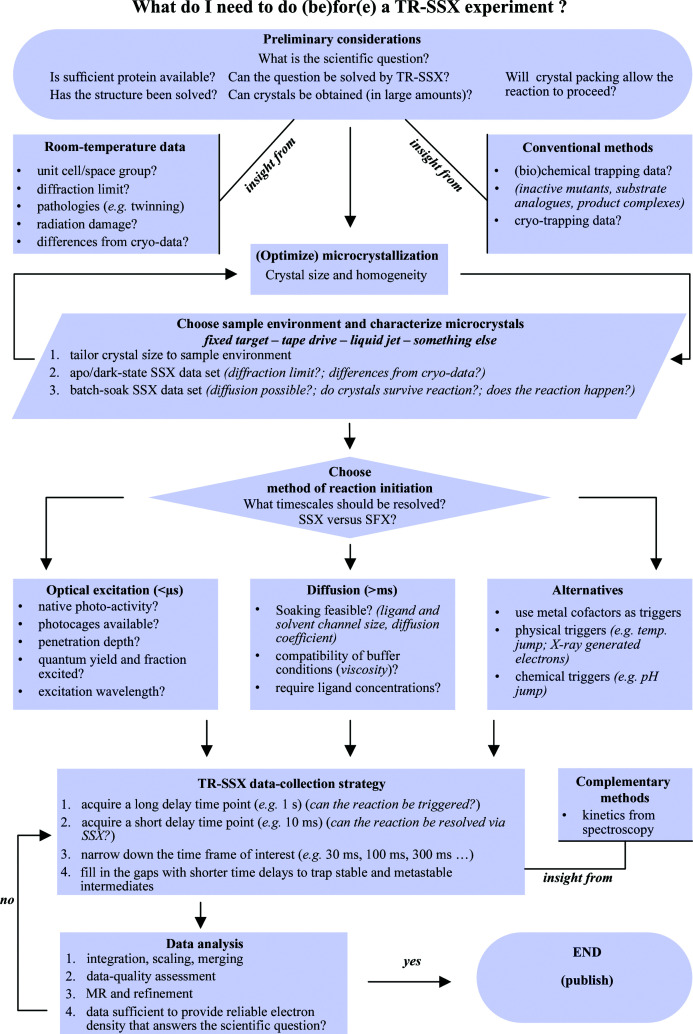
A workflow scheme for time-resolved serial synchrotron crystallography experiments.

Despite this increasing interest in time-resolved techniques in the past decade, the number of groups working on developing new methodologies remains small and the newly developed tools and techniques are currently mainly only accessible to experts in the field. In addition, in contrast to collecting static data from single crystals, time-resolved crystallography experiments require more careful planning and preparation to obtain successful results. As time-resolved crystallography methods continue to further evolve they will of course undergo simplification and automation and thus become more accessible to a larger user base. However, as this is not yet the case, in this article we aim to provide new users, who are already familiar with classical crystallographic approaches, with a systematic and didactic approach to preparing for and carrying out a time-resolved serial synchrotron crystallographic (TR-SSX) experiment (Fig. 1 ▸). Similar considerations are also required for SFX experiments, although these are not the focus of this review. We also highlight some of the common problems that we have encountered so far and how we have adjusted our approach to address these.

## 2. Preliminary considerations

Ultimately, the design of any time-resolved experiment depends on the scientific question that is being asked, the experimental data that already exist and whether the required experiment is, in principle, feasible.

### 2.1. The scientific question

We include an explicit consideration of the scientific question being asked as, while it is always tempting to ‘do what you know’, in some cases the question being posed can be answered much more easily with a noncrystallographic approach. Classical biochemical methods such as kinetics and spectroscopy, or alternate biophysical methods such as magnetic resonance and/or fluorescence techniques, can provide a considerable degree of insight into mechanisms of ligand binding, reaction turnover and protein conformational change and can often be performed in-house. SAXS and single-particle electron cryo-microscopy also provide structural data and are particularly well suited to the study of systems where large structural changes are expected. Finally, the tried and tested structural enzymology approaches of cryo-trapping and mechanistic trapping are extremely powerful and it is well worth testing whether such an approach can already answer the question at hand. The adage ‘choose your weapon wisely’ should be taken to heart here.

### 2.2. Feasibility

In spite of many recent improvements, serial crystallo­graphy experiments are still very sample-demanding, and this is even more the case for time-resolved studies where several structures are to be obtained, each of which requires at least ∼5000 diffraction patterns (Moreno-Chicano *et al.*, 2019 ▸; Weinert *et al.*, 2017 ▸; Mehrabi *et al.*, 2021 ▸; Gorel *et al.*, 2021 ▸). Thus, a critical question is whether a sufficient amount of protein is available. The protein should also crystallize readily in order to yield a sufficient supply of reproducible crystals. As in any crystallographic project, the crystals must also diffract sufficiently well to answer the scientific question being posed. Capturing changes between different low-resolution (<3 Å) structures will primarily yield data pertaining to gross protein motions. However, observing the intricacies of key biochemical events, such as conformational changes in the protein or ligands, water-network alteration, bond stretching and bond breakage/formation, requires near-atomic resolution. Finally, prior to any time-resolved study, reference structures of the protein should be determined, ideally by SSX at room temperature, in order to assess whether the crystal packing will allow the reaction to proceed. Surprisingly large conformational changes have been reported to be accommodated by macromolecular crystals, but this is not a given and should be tested (Ramakrishnan *et al.*, 2021 ▸; Harata & Akiba, 2006 ▸; Stagno *et al.*, 2017 ▸). In addition, in some cases crystal packing can block access to the active site and in this case new crystallization conditions that result in a different packing are likely to be required.

### 2.3. Prior experimental knowledge

The question of how many preliminary data should be obtained before embarking on a time-resolved experiment is an important one that requires balancing the effort and time needed to generate the preliminary data with how much the data will help to streamline and optimize the time-resolved experiment. In our experience, however, this is always effort well spent.

#### 2.3.1. The crystals

A good understanding of the crystal system will greatly enhance the likelihood of success of a TR-SSX experiment. For example, information on differences in the behaviour of the crystals at room temperature and 100 K, such as unit-cell changes or conformational differences, is important for optimal data processing and subsequent electron-density interpretation. A well recorded, high-quality single-crystal data set is also invaluable here. Any crystal pathologies, such as twinning, are also best identified in single crystals before any serial data acquisition. If crystal pathologies are present, such as perfect twins or unresolvable indexing ambiguities, then new crystal conditions are needed before further progress can be made.

#### 2.3.2. Soaks and trapped states

Mechanistically and cryo-trapped structures almost always provide valuable insight into (meta)stable intermediates that will aid in the interpretation of the data derived from the TR-SSX experiments. Such structures can be as simple as a substrate, product or inhibitor soak, or can be from mutant variants of the protein of interest. Differences between these trapped states and similar intermediates observed in the time-resolved data can provide important information about the reaction mechanism.

#### 2.3.3. Reaction kinetics

Last, but very much not least, some insight into the reaction kinetics, ideally in the crystals themselves or under the crystallization buffer conditions, should be available. A convenient source to quickly obtain information on protein kinetics is the BRENDA database (https://www.brenda-enzymes.org; Chang *et al.*, 2021 ▸). This information helps both with decisions on how to best initiate the reaction and the choice of time points to be measured.

## 3. Sample preparation

### 3.1. Microcrystal properties: size and homogeneity

Different sample-delivery and reaction-initiation methods have different crystal size requirements, and thus the crystal size has to be tailored to the planned experiment. While fixed-target and tape-drive approaches are relatively flexible with respect to crystal size, liquid-jet methods usually require smaller crystals (<10 µm; Mehrabi *et al.*, 2020 ▸; Beyerlein *et al.*, 2017 ▸; Martiel *et al.*, 2019 ▸). The crystal size also directly affects the penetration depth of optical triggers and the diffusion time in mixing approaches (Mehrabi, Schulz, Agthe *et al.*, 2019 ▸; Schmidt, 2013 ▸; Grünbein *et al.*, 2020 ▸). It is also important that the microcrystals are as homogenous as possible. This is important in ensuring that the reaction initiation is similar for all crystals and also improves the downstream processing of the diffraction data.

### 3.2. Obtaining microcrystals

**Figure 2:**
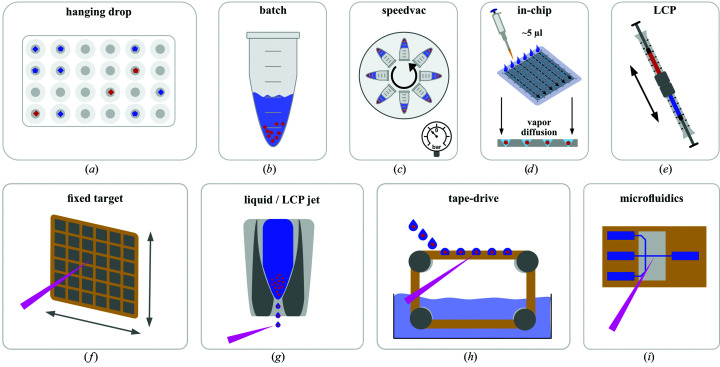
Overview of commonly used microcrystallization techniques and SSX sample environments. Top: commonly applied microcrystallization approaches include (*a*) hanging-drop or sitting-drop crystallization, (*b*) batch crystallization, (*c*) vacuum-induced (batch) microcrystallization, (*d*) in-chip microcrystallization based on vapour-diffusion protocols and (*e*) LCP-based microcrystallization. Bottom: commonly used sample environments for serial synchrotron crystallography are (*f*) fixed-target-based approaches, (*g*) liquid or viscous jets, (*h*) hybrid methods such as tape-drive sample delivery and (*i*) microfluidic systems.

The crystallization conditions for previously obtained single crystals are an excellent starting point for obtaining microcrystals. Usually, these crystallization conditions can be tweaked to increase nucleation to yield more, smaller crystals. Some of the common methods for obtaining microcrystals will briefly be discussed here (Fig. 2 ▸).

#### 3.2.1. Mechanical disruption

The simplest and quickest method to obtain a slurry of microcrystals and even nanocrystals is to apply mechanical force. This is typically performed using large single crystals obtained by standard hanging-drop or sitting-drop methods, isolating these crystals in sufficient number, adding glass beads and vortexing. The glass beads crush the large crystals and, depending on how long the sample is vortexed for, result in different size distributions of crystals. This method has been used to obtain nano-sized or near-nano-sized crystals for use in gas dynamic virtual focusing nozzle (GDVN) liquid jets at XFELs or for serial electron diffraction (Bücker *et al.*, 2020 ▸; Cheng, 2020 ▸). The primary drawbacks of this method are that the shearing and crushing forces applied to the crystals can result in mechanical disruption of the crystal lattice, reducing the diffracting power, and that it usually results in a quite heterogeneous crystal size distribution that can require large crystal fragments to be removed by filtration.

#### 3.2.2. Mass vapour-diffusion crystallization

Modifying the starting vapour-diffusion conditions, usually by increasing the precipitant or protein concentration, can result in reasonably sized microcrystals using standard vapour-diffusion methods. Subsequently, crystals from hundreds of hanging or sitting drops must be pooled together for use at the beamline. While this approach does not require any specialist hardware beyond that found in a normal crystallography laboratory, it is extremely tedious, and the harvesting and pooling of drops can be detrimental to crystals which are sensitive to handling.

#### 3.2.3. Batch crystallization

Batch methods, in which large volumes (usually >50 µl) of both mother liquor and protein solution are mixed in a vial, are ideal for obtaining large numbers of homogenously sized microcrystals. Recently, a number of systematic approaches for obtaining microcrystals via batch-crystallization methods using different precipitation agents have been described (Beale *et al.*, 2019 ▸; Stohrer *et al.*, 2021 ▸). An extension to batch crystallization takes advantage of rapid evaporation from a vacuum environment (Martin & Zilm, 2003 ▸). This method was initially used to obtain nanocrystals for use in solid-state NMR and has two major advantages: (i) placing the mixed solution under vacuum results in very rapid nucleation, and thus microcrystals can usually be obtained within 10–20 min, for example immediately prior to data collection, and (ii) because nucleation occurs in a very short time frame, the resulting crystals generally have a homogeneous size distribution.

#### 3.2.4. On-target crystallization

If fixed targets are used for sample delivery, then as an alternative to batch crystallization, crystals can be grown directly on the fixed target. Here, the protein and mother liquor are mixed and applied directly onto the chip surface. If the sample is viscous it can be forced into the features of the chip by drawing a razor blade across the chip surface. Crystals are then grown using a vapour-diffusion process very similar to the hanging-drop method (Lieske *et al.*, 2019 ▸; Norton-Baker *et al.*, 2021 ▸). In addition to allowing direct transfer of the starting vapour-diffusion conditions, this approach completely avoids crystal handling, produces a very homogenous crystal size distribution and has the lowest sample consumption of all of the methods described here. Single data sets can be obtained from only micrograms of protein (Norton-Baker *et al.*, 2021 ▸).

#### 3.2.5. Seeding

If obtaining either the desired crystal size or a homogenous distribution is difficult, microcrystal seeding in batch is a powerful approach. By using relatively high concentrations of seeds, high yields of microcrystal crystals of the desired size and low size heterogeneity can be obtained.

### 3.3. Initial crystal characterization

#### 3.3.1. Diffraction quality

To determine whether microcrystals are suitable for a TR-SSX experiment, minimally a static data set should be acquired. This can be performed simply using grid or mesh scans on a spine loop both at cryo and room temperatures to determine the resolution limits and general diffraction properties, ideally using the same beam parameters as will be used for the time-resolved experiment. The focus of this characterization should of course be on the room-temperature data. If possible, it is also advisable to test the full crystal-handling workflow planned for the time-resolved experiment, for example chip-loading conditions, liquid-flow parameters in microfluidic devices or jets *etc.*, in order to avoid any surprises during the actual experiment.

Obtaining suitable microcrystals for time-resolved studies often requires iterative optimization of both the crystal-growth conditions and the crystal handling (including sample delivery). An important parameter that must not be neglected is the humidity, as slight changes in hydration both improve and obliterate diffraction (Bowler *et al.*, 2006 ▸; Sanchez-Weatherby *et al.*, 2009 ▸; Russo Krauss *et al.*, 2012 ▸).

#### 3.3.2. Turnover

An important final control before the time-resolved experiment itself is a simple batch soak (for long enough for the reaction to complete) of the microcrystals with the substrate/ligand of interest or, for photocaging experiments, illumination, which allows the quick assessment of a number of important parameters. Do the microcrystals survive exposure to the ligand or optical trigger? Are diffraction properties affected? Does the reaction actually take place in the microcrystals? If the answer to any of these questions is ‘no’ then a rethink of the experimental plan is needed.

## 4. Sample delivery

Serial crystallography approaches at both XFELs and synchrotrons generally encompass four types of sample-delivery methods, which include liquid-injection, microfluidics, fixed-target and hybrid approaches; these have recently been reviewed in detail (Martiel *et al.*, 2019 ▸; Grünbein & Nass Kovacs, 2019 ▸). We will therefore limit the discussion here to sample environments which have successfully been used for time-resolved data collection at synchrotrons (Fig. 2 ▸). Assuming that the crystals are all well diffracting, isomorphous and randomly oriented with respect to the X-ray beam, the hit rate is the major consideration when choosing a sample-delivery method.

### 4.1. Liquid and viscous jets

The first serial sample-delivery devices employed at XFELs were liquid-injection devices using a GDVN. Due to their speed, GDVN injectors are well suited to XFEL experiments but are less ideal for synchrotron measurements, especially on monochromatic macromolecular crystallography (MX) beamlines. Similar devices have, however, been developed to extrude viscous media such as LCP; these deliver sample more slowly and are ideal for the longer exposure times required at synchrotron sources (Martiel *et al.*, 2019 ▸). These can be combined with photoactivation for time-resolved experiments (Weinert *et al.*, 2019 ▸).

### 4.2. Fixed targets

Fixed-target approaches generally come in one of two flavours, either using spine mounts with mesh loops or one of several different solid-support solutions (Martiel *et al.*, 2019 ▸).

#### 4.2.1. Mesh loops

The simplest fixed-target approach involves loading a mesh loop with a crystal slurry and either performing a simple grid scan, a helical scan or a *MeshAndCollect*-type data collection (Zander *et al.*, 2015 ▸). While quick and simple, this approach generally requires cryo-conditions and is not amenable to either mixing/diffusion or photoactivation.

#### 4.2.2. Solid supports

A number of solid-support designs have been reported that can be raster-scanned through the X-ray beam either using a modified goniometer (for example Roadrunner) or using (piezo-driven) three-axis stages (for example HARE/Oxford photochips) (Mehrabi *et al.*, 2020 ▸; Oghbaey *et al.*, 2016 ▸; Zarrine-Afsar *et al.*, 2012 ▸; Sherrell *et al.*, 2015 ▸; Roedig *et al.*, 2017 ▸). The advantage of solid supports are a lower sample consumption relative to liquid-jet approaches (Norton-Baker *et al.*, 2021 ▸; Mehrabi, Schulz, Agthe *et al.*, 2019 ▸; Lieske *et al.*, 2019 ▸) and a relatively high hit rate, which can be up to 100% if crystal locations can be mapped prior to data collection (Oghbaey *et al.*, 2016 ▸). Additionally, many of the fixed-target solutions can accommodate a wide range of crystal sizes and morphologies without modification of the support. This allows easy screening of different samples or different crystals of the same sample within a single experiment.

Currently, the HARE/Oxford photochips are the only fixed-target chips that are routinely used for time-resolved measurements at both XFELs and synchrotrons (Schulz *et al.*, 2018 ▸; Mehrabi, Schulz, Agthe *et al.*, 2019 ▸; Mehrabi, Schulz, Dsouza *et al.*, 2019 ▸). Both the HARE and Oxford photochips are amenable to experiments involving photoactivation or mixing/diffusion using the LAMA approach (Mehrabi, Schulz, Agthe *et al.*, 2019 ▸). Because the features on these chips are well ordered and each position is known, crystals can be imaged more than once, allowing unique data-acquisition schemes such as the Hit-And-REturn (HARE) approach (Schulz *et al.*, 2018 ▸). This method allows data acquisition with a wide range of delay times from a few milliseconds to many seconds, while keeping the total data-collection times per chip low. A combination of the HARE and LAMA approaches is currently the primary method of choice at the T-REXX endstation (Beamline P14.EH2) at PETRA III, which is the world’s first dedicated endstation for time-resolved serial synchrotron crystallography.

#### 4.2.3. Microfluidic devices

Microfluidic devices, such as 3D-MiXD, employ simple 3D-printed designs with multiple channels controlled by a series of syringe pumps where crystals and substrate can be mixed before flowing into the X-ray beam path for imaging (Monteiro *et al.*, 2020 ▸). They are simple to set up, run reliably for many hours and are compatible with both photoactivation and rapid mixing, and their sample consumption is ∼50 µl h^−1^ (Monteiro *et al.*, 2020 ▸). While some shortcomings do exist with microfluidic devices, such as clogging due to bubble formation or fouling of the Kapton windows, these can largely be avoided with the aid of a fast shutter.

#### 4.2.4. Hybrid approaches

Hybrid approaches, such as the mix-and-diffuse or drop-on-tape approaches, employ a moving thin Kapton film onto which droplets containing crystals are deposited before being carried through the X-ray path (Roessler *et al.*, 2013 ▸; Butryn *et al.*, 2021 ▸; Beyerlein *et al.*, 2017 ▸; Fuller *et al.*, 2017 ▸). The drop-on-tape method uses a combination of microfluidics to mix both crystals and substrate before a nozzle deposits small droplets onto the Kapton film. The mix-and-diffuse approach uses piezo transducers to eject droplets of crystal-containing solution onto the tape. In both cases, before arriving at the X-ray beam, the tape can carry the droplets through activation regions where they can interact with, for example, lasers or different atmospheres.

## 5. Reaction initiation

All time-resolved crystallography experiments use the pump–probe approach in which a reaction is initiated (pumped) in a crystal, which is then probed, after a defined time delay, by X-rays to yield a diffraction pattern. A key aspect of the experimental design is therefore how the reaction will be initiated. Important here is that a sufficient fraction of molecules in the crystal are activated as synchronously as possible to ensure that the resulting time-dependent electron-density maps can be interpreted and that the temporal resolution is appropriate to the timescale being probed. We note here that many enzymes and biomolecular processes involving conformational changes are in fact relatively slow, with median turnover rates of the order of *k*_cat_ = 13.7 s^−1^ (Bar-Even *et al.*, 2011 ▸). These timescales are ideally suited for study by time-resolved SSX.

### 5.1. Accessible timescales are a function of both the X-ray source and the reaction-initiation method

#### 5.1.1. X-ray source

The minimal time needed to record a usable diffraction pattern from a macromolecular microcrystal is a function of the brightness of the X-ray beam. Third- and fourth-generation monochromatic microfocus synchrotron MX beamlines can reach a time resolution of at least 100 µs, and with multiplexing methods such as HATRX can, in principle, access nanosecond timescales, although this has yet to be experimentally demonstrated (Yorke *et al.*, 2014 ▸). Laue crystallography beamlines can achieve a time resolution of 100 ps. Sub-100 ps experiments are the preserve of XFELs, which can even address events on the order of tens of femtoseconds (Chapman, 2019 ▸). For time-resolved structural biology the availability of suitable X-ray sources is no longer a limiting factor.

#### 5.1.2. Reaction-initiation method

The time required for reaction initiation is the true limitation on the timescales that can be probed by time-resolved crystallography. For systems where the reaction can be triggered by photoisomerization or direct photocleavage, femtosecond timescales are accessible. However, when a series of dark reactions must occur after the initial photoexcitation before the system becomes fully active, or where initiation is by rapid mixing, the accessible timescales become much longer.

### 5.2. Photoactivation

#### 5.2.1. Photocages

As only a fraction of proteins are naturally photosensitive (<0.5%; Monteiro *et al.*, 2021 ▸), caged compounds are an attractive option for reaction initiation in synchrotron-based time-resolved experiments, where the timescales of interest are usually >100 µs. Either a ligand or the protein can be made photosensitive by the addition of a photolabile protecting group, which can then be released with a laser pulse. The photocaged moiety should be inert and, if a small molecule has been caged, can be co-crystallized or soaked into the crystal prior to X-ray measurements. Selection of the optimal photocaging group for a particular experiment requires the consideration of a number of parameters, and these are discussed extensively in the recent review by Monteiro *et al.* (2021 ▸). A key consideration is the decaging rate, *i.e.* how quickly the uncaged, active state of the system is achieved. Obviously, this must be faster than the reaction steps to be studied in the time-resolved experiment. It is not always appreciated how slow decaging can be. While pH jumps using photoacids can occur on sub-microsecond timescales, many cages release in the millisecond time regime. The concentration of the caged compound should also be at least on parity with the concentration of the protein in the crystal, and ideally higher. For experiments using photoactivation, care must be taken when dealing with lasers of high power densities to avoid artefacts which can arise due to multiphoton effects; this is discussed in detail by Grünbein *et al.* (2020 ▸).

#### 5.2.2. *T*-jumps

Excitation of the water within a crystal with an IR pulse can result in a temperature jump of several kelvin within a few nanoseconds as the energy is rapidly transferred from the water to the proteins. This can be sufficient to trigger reaction turnover, but has so far seen most application in SAXS/WAXS experiments rather than in TR-SSX (Levantino *et al.*, 2015 ▸).

### 5.3. Diffusion or rapid mixing

While the diffusion times of small-molecule ligands into macroscopic crystals can be of the order of several seconds, the situation is quite different in microcrystals. Due to the surface-to-volume ratio, the diffusion time accelerates by two orders of magnitude when the crystal volume is reduced by only one order of magnitude; this relationship gives rise to low-millisecond diffusion times for microcrystals with dimensions of a few micrometres (Schmidt, 2013 ▸; Mehrabi, Schulz, Agthe *et al.*, 2019 ▸; Butryn *et al.*, 2021 ▸; Beyerlein *et al.*, 2017 ▸; Schmidt, 2020 ▸). This means that mixing/diffusion-based methods not only become viable but are an excellent and simple method for reaction initiation in time-resolved SSX experiments.

In general, mixing methods require a simpler hardware setup compared with photoexcitaton approaches and circumvent many of the difficulties associated with, for example, multiphoton effects. Nevertheless, some thought is still required when using mixing to initiate the reaction. As for photoactivation, the turnover rate of the target protein should be lower than the time required for the ligand to diffuse to the centre of the crystal. This critical depth parameter is a function of the kinetic properties of the enzyme and the diffusion coefficient of the ligand (Makinen & Fink, 1977 ▸). The diffusion coefficient can vary greatly depending on the size and hydrophobicity of the ligand, the viscosity of the crystallization mother liquor and the size of the solvent channels within the crystal. As with caged compounds, the concentration of the ligand should be at least that of the protein in the crystal. In practice it is advisable to (greatly) exceed this concentration and work with the maximum concentration possible. This may require some screening of crystallization mother liquors to identify conditions which maintain the crystal while also allowing ligand solubilization at high concentrations.

Many proteins can be activated via secondary triggers, such as cations, which are required for protein function (Richter, 2013 ▸). This can be a convenient and elegant workaround to achieve rapid and homogeneous activation as diffusion of ions is quick and crystallizing a protein–ligand complex may prove to be easier than the protein alone.

## 6. Choice of time points

### 6.1. Crystals versus solution

The crystal environment can alter the kinetics of the system compared with those measured in dilute solution, and in extreme cases can result in deviations into dead-end or non­physiological pathways (Konold *et al.*, 2020 ▸; Makinen & Fink, 1977 ▸). A simple test here is to remeasure the solution-phase kinetics but using a buffer system as close to the crystallization conditions as possible. Alternatively, microspectrophotometry can be used to directly follow the reaction progress in the microcrystalline slurry and precisely define the timescales of interest (Pearson *et al.*, 2004 ▸; Wilmot *et al.*, 2002 ▸; Pearson & Mozzarelli, 2011 ▸).

### 6.2. Shoot it and see?

If no data on the kinetics in the crystalline state are available, the simplest data-collection strategy, albeit high-risk, is to use the existing catalytic timescales obtained from solution measurements and observe whether intermediates obtained from the TR-SSX measurement agree. However, this can be risky, especially when beamtime is limited. We therefore suggest instead initially measuring one long time delay (for example >1 s) to determine whether the reaction has run to completion in the crystal. Next, one short delay (for example 10–20 ms) should be recorded to test whether the reaction is slow enough to be resolved by TR-SSX, *i.e.* whether the end state has already been reached or not. This approach makes good use of beamtime and allows mapping of the temporal boundary conditions.

Following this, a simple data-collection strategy can be employed whereby series of initially short time delays are measured (10, 50, 100 ms …) with subsequent time delays becoming longer, representing a pseudo-logarithmic timescale that attempts to capture the entirety of the catalytic cycle. For mixing/diffusion-based approaches the initial time point that can be collected can be estimated based on knowledge of the crystal size and the expected or measured diffusion times. Following analysis of the data obtained, further measurements can fill in the gaps around key events.

### 6.3. Data-collection modes

#### 6.3.1. Classical pump–probe

**Figure 3:**
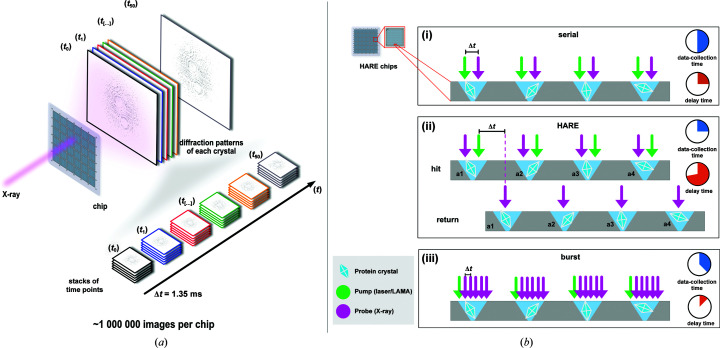
(*a*) Schematic representation of burst data collection. (*b*) Visual representation of various data-collection schemes on the HARE chip from standard pump–probe to the Hit-And-Return (HARE) approach to burst data collection.

Pump–probe experiments display the simplest data-collection strategy: an individual crystal is ‘pumped’ via some trigger, that is the reaction is initiated, and after a desired delay time has been achieved the crystal is ‘probed’ via the X-ray pulse. This is the standard scheme for acquiring time points with a short time delay (<100 ms; Fig. 3 ▸ *b*, i).

#### 6.3.2. Burst pump–probe

In a burst experiment, taking advantage of high-repetition-rate detectors, each crystal is pumped and a sequential series of diffraction images are acquired with a Δ*t* equaling the repetition rate of the detector before moving to the next crystal. The images are then stacked according to their respective time points (Ebrahim *et al.*, 2019 ▸; de la Mora *et al.*, 2020 ▸; Figs. 3 ▸ *a* and 3 ▸ *b*, iii).

#### 6.3.3. HARE

The Hit-And-REturn (HARE) approach allows the collection of long time delays without the large increase in total data-collection time for a chip that would otherwise result from a standard pump–probe measurement with serial data collection (Schulz *et al.*, 2018 ▸). Unlike a standard pump–probe experiment, in a HARE experiment a series of crystals are first pumped and subsequently probed in the same order. This means that the HARE method enables collection of time delays that can span up to several minutes, while the total data-collection time for a chip remains the same (Fig. 3 ▸ *b*, ii).

## 7. Data-quality assessment

Obtaining high-quality data should be the goal when planning the TR-SSX experiment, and rapid feedback on the progression of the experiment in terms of hit rates, average resolution and ideally electron-density maps is invaluable in order to help to determine when sufficient data have been collected for each time point (Basu *et al.*, 2019 ▸; Beilsten-Edmands *et al.*, 2020 ▸; Ke *et al.*, 2018 ▸; Barty *et al.*, 2014 ▸; White *et al.*, 2016 ▸). Good-quality data are especially important for time-resolved studies, where dynamic and low-population states can complicate the interpretation of electron-density maps. Time-resolved XFEL-based experiments, which generally involve femtosecond to nanosecond timescales, require a more rigorous treatment of the experimental setup, especially in terms of timing tools and in terms of processing and analyzing XFEL data, where careful validation is required to avoid overestimating the small structural changes occurring on ultrafast timescales. The excellent recent review by Gorel and coworkers extensively discusses the best practices for XFEL-based experiments, many of which also hold true for synchrotron-based experiments (Gorel *et al.*, 2021 ▸).

### 7.1. How many data are enough?

Arguably the first consideration when assessing the quality of any data set should be completeness; this single parameter is generally applicable to any crystallographic experiment and provides context for all other data-quality parameters. If the completeness of the data set is below an approximate cutoff of 95% then the meaning that can be derived from the scaling statistics is limited (Mehrabi *et al.*, 2021 ▸). The pertinent question when collecting data may then appear to be ‘how many diffraction images do I need to collect to obtain a complete data set?’. However, particularly in a time-resolved experiment, completeness alone is not enough. The data should also be of high redundancy in order to ensure stable scaling and reliable error estimation.

The amount of data needed is ultimately dependent on both the sample and the experimental design. Although the minimum reported value for a good-quality data set is ∼5000 indexable diffraction patterns, this number certainly increases the less populated the state one wants to observe is (Weinert *et al.*, 2017 ▸; Moreno-Chicano *et al.*, 2019 ▸; Mehrabi *et al.*, 2021 ▸). Therefore, in order to determine when an experiment is complete, it is beneficial to monitor the hit rate (*i.e.* the ratio of indexable diffraction patterns to the number of X-ray pulses), scaling statistics (such as CC_1/2_ or *R*_split_), completeness, redundancy and resolution.

Prior knowledge of the space group and unit cell of the crystal are also essential when carrying out time-resolved experiments to help to avoid indexing ambiguities and support automatic data processing. In principle, higher symmetry point groups require fewer images for a complete data set (Dauter, 1999 ▸). However, the need for high redundancy and reliable error estimate remain and so in practice there is no real reduction in the number of diffraction patterns that are needed. On-the-fly data-processing methods utilizing the computational parallelization available at XFEL and synchrotron sources to allow the progress of the experiment to be monitored in real time are key to ensuring that data-collection strategies optimize data quality and experimental efficiency (Basu *et al.*, 2019 ▸; Beilsten-Edmands *et al.*, 2020 ▸; Ke *et al.*, 2018 ▸; Barty *et al.*, 2014 ▸; White *et al.*, 2016 ▸). The hit rate of the experiment is a useful metric to give an indication of how many runs of the experiment will be required, but measuring this single metric does not indicate the completeness of a data set, only the efficiency of the delivery method.

### 7.2. Preferred orientation

In both fixed-target and flow-based sample-delivery approaches the crystal morphology can cause crystals to adopt a preferred orientation with respect to the X-ray beam. If this occurs it can be problematic, as serial crystallographic delivery systems are generally not designed to allow reorientation of the sample. Real-time feedback about the completeness and multiplicity of a data set can provide an early indication of this issue (Mariani *et al.*, 2016 ▸), and methods have been developed to encourage random orientation in jets and fixed-target chips (Schlichting, 2015 ▸; Murray *et al.*, 2015 ▸). Utilizing microfluidic and acoustic drop injection techniques can be useful in such cases (Zhao *et al.*, 2020 ▸; Davy *et al.*, 2019 ▸).

### 7.3. Isomorphism

The requirement that every crystal in a particular sample is isomorphous should not be taken lightly, as the presence of non-isomorphism can obscure the presence of weakly bound ligands and states with low populations. Separating data by grouping different isomorphs according to the unit-cell dimensions, cross-correlation statistics or by utilizing genetic algorithms can help to address this issue (Foadi *et al.*, 2013 ▸; Giordano *et al.*, 2012 ▸; Santoni *et al.*, 2017 ▸; Zander *et al.*, 2016 ▸; Ginn, 2020 ▸).

It should also be noted that both laser- and diffusion-based initiation can have a profound effect on the isomorphism of the sample. Changes in unit-cell size and a gradual decline in diffraction quality may be observed, especially at longer time delays, due to a progressive increase in the disorder of the crystalline lattice and an increase in the superposition of intermediate states. Large amounts of energy are deposited into the crystal during laser initiation, and changes in temperature and pH can impact not only the diffraction quality but also the kinetics of the reaction that is being followed (Grünbein *et al.*, 2020 ▸).

### 7.4. Resolution

Resolution should be considered in terms of the minimum resolution necessary to reliably identify the important structural changes associated with the reaction of interest. A large protein conformational shift may be apparent at ∼3 Å resolution; however, a higher resolution is needed if conclusions are to be drawn about bond formation/breakage, backbone flips, rearrangement of solvent molecules, oxidation state and isomerization, where small changes to specific interatomic distances are integral to the structural change being monitored.

### 7.5. Scaling statistics

In a single-crystal experiment *R*_meas_, 〈*I*/σ(*I*)〉 and completeness are the most commonly reported statistics, and a number of rules of thumb relating to ‘acceptable’ values of *R*_meas_ and *I*/σ(*I*) have emerged (Karplus & Diederichs, 2012 ▸) that are not necessarily directly applicable to serial data.

*R*_meas_ measures the spread of recorded intensities for a particular reflection around the average intensity, with a correction to account for redundancy. Since the strength of diffraction data decreases with resolution, a resolution cutoff point is often defined where *R*_meas_ reaches a value of approximately 0.6 and 〈*I*/σ(*I*)〉 is approximately equal to 2. The rationale behind these cutoff values was to ensure that weaker, noisy data are not included in structure refinement. However, with the almost ubiquitous use of photon-counting detectors the justification for excluding weak data [as defined by 〈*I*/σ(*I*)〉] has become almost obsolete (Evans & Murshudov, 2013 ▸). In addition, 〈*I*/σ(*I*)〉 as an indication of the signal-to-noise ratio (SNR) is not always reliable due to the various ways in which σ(*I*) may be calculated.

#### 7.5.1. *R*_merge_ and *R*_split_

*R*_merge_ is particularly problematic as a data-quality indicator for serial data, since serial data reduction relies on sampling large distributions of intensities. In serial data the spread of intensities is large by design and so this metric provides no useful indication of data quality (White *et al.*, 2012 ▸). In the case of serial data *R*_merge_ is better replaced by *R*_split_, where the data are split into two equal (and random) groups and the agreement between the intensities in each list is compared and corrected for multiplicity.

#### 7.5.2. CC_1/2_

The half-data correlation coefficient CC_1/2_ is widely reported for serial data as it provides a useful metric for determining a suitable resolution cutoff and for identifying rogue images (Assmann *et al.*, 2016 ▸; Karplus & Diederichs, 2015 ▸). Here the data are split into two randomly selected groups, or upwards of ten resolution shells, and the Pearson correlation coefficient is calculated between the average intensities in each subset. This method has the advantage of giving a clear indication regarding the precision of a data set with values ranging from 1 (perfectly correlated, precise) to 0 (no correlation, imprecise) (Assmann *et al.*, 2016 ▸).

In summary, it is unwise to rely on a single scaling statistic to guide data-collection strategies. *R*_split_, 〈*I*/σ(*I*)〉 and CC_1/2_ each provide useful insight into the overall quality of the model and should all be taken into account (Wang, Brudvig *et al.*, 2017 ▸).

### 7.6. Electron density

Although scaling statistics provide an indication of the overall quality of the data sets for each time point, they do not provide information about the structure or the actual reaction being investigated. Only by inspection of the electron density of each individual data set is it possible to confirm the presence of a ligand or intermediate or the successful initiation of a reaction in the crystal. Although the use of complementary spectroscopic methods can aid with monitoring these changes in real time, a spectroscopic signal is no guarantee that the structural changes will be discernible in the electron density.

## 8. Radiation damage

### 8.1. Minimum exposure time

For a TR-SSX experiment the minimum exposure time to allow the required resolution to be achieved, whilst also ensuring that the data can be indexed and integrated reliably, should be determined before the time-resolved experiment begins. This is important to minimize the effects of radiation damage on the data.

### 8.2. Dose limits for room-temperature SSX

The radiation tolerance and the available dose budget for SSX experiments at room temperature has recently been addressed by de la Mora and coworkers, who systematically compared serial data collection from lysozyme crystals at cryo and room temperatures. They established a global damage dose limit of 15.3 MGy at 100 K and 0.57 MGy at room temperature (de la Mora *et al.*, 2020 ▸). However, specific radiation damage, which could obscure or even initiate catalytic processes, is a greater concern in time-resolved studies, and they suggest keeping the cumulative dose per crystal below 80 kGy. This limit may be even lower if the system being investigated contains metal centres or redox-active cofactors that can be damaged even at very low doses. These dose limits can be directly translated into the number of exposures that a crystal can tolerate in an SSX burst experiment and thus put a boundary on the maximal achievable number of images per crystal.

### 8.3. Monitoring radiation damage

In the cases of proteins containing metal centres, chromophores and photocaged ligands site-specific damage is an extremely important consideration, although there is evidence that global and specific radiation damage are ‘re-coupled’ at room temperature and that specific damage may therefore be less of a concern than initially feared (Gotthard *et al.*, 2019 ▸). Real-time monitoring of the sample by complementary methods such as UV–Vis and Raman spectroscopy can help to identify X-ray-induced changes to the structure that may not be linked to the reaction of interest.

**Figure 4:**
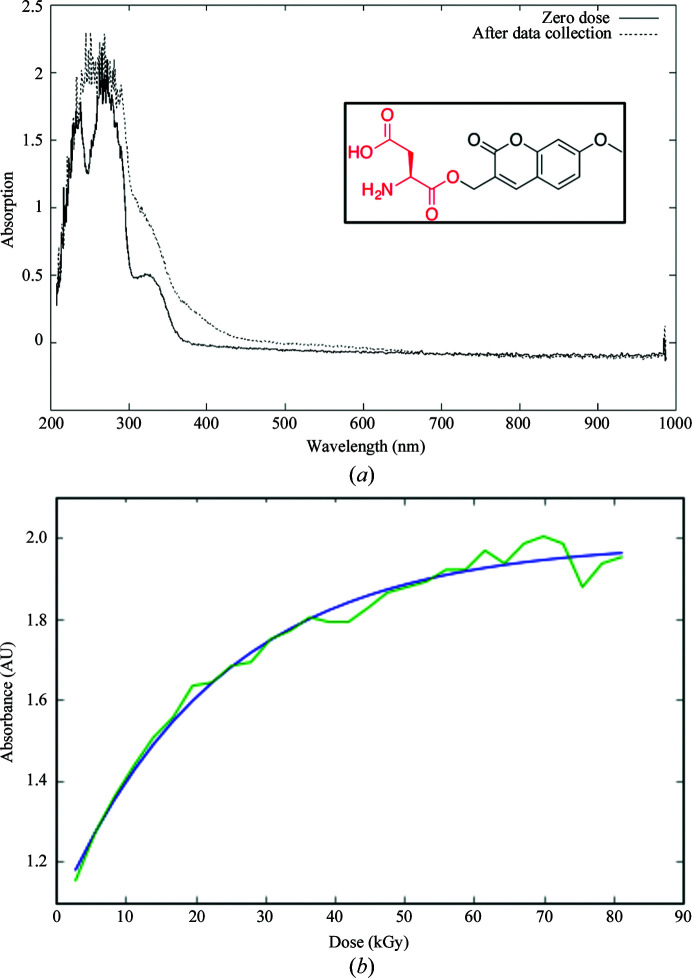
Online single-crystal UV–Vis spectroscopy showing the decaging of a coumarinyl photocage at 100 K in the absence of laser initiation, recorded on FIP(BM30A) at the ESRF. (*a*) UV–Vis spectra recorded from the same crystal, in the same orientation, before and after X-ray data collection show significant changes to the absorption spectrum of aspartate decarboxylase (ADC) crystals containing (coumarin-4-yl)-methyl-caged l-aspartate (inset) before (solid line) and after (dotted line) X-ray data collection. (*b*) Monitoring of the absorption of ADC containing (coumarin-4-yl)-methyl-caged l-aspartate at 350 nm during continuous X-ray exposure at 100 K (data shown in green with a fitted single exponential in blue) shows that photolysis is almost complete after a total absorbed dose of 80 kGy (*RADDOSE*-3*D*; Zeldin *et al.*, 2013 ▸). The contribution of the UV–Vis light source cannot be ruled out: no significant spectral changes were observed in the absence of X-radiation (B. A. Yorke, M. Sans Valls, J. I. Zaitseva-Kinneberg, D. Von Stetten and A. R. Pearson, personal communication).

In experiments using photocages where a ‘dark’ image is recorded from a crystal before photoactivation, spectroscopic measurements are also important to confirm that decaging only occurs upon laser illumination. There is some evidence indicating that photocleavage of caged ligands may be initiated by X-rays as well as UV light (Fig. 4 ▸; Specht *et al.*, 2001 ▸). However, there are also cases where the photocaged group is clearly unaffected by X-ray exposure, suggesting that the susceptibility of the photocage to X-rays may be modulated by the buffer conditions, and by the local environment in cases where the photocaged compound is specifically bound within the crystal (Kesgin–Schaefer *et al.*, 2019 ▸).

## 9. Assessing low-populated states

Electron-density maps derived from time-resolved structural data contain a superposition of unreacted and reacted molecules, especially at longer time points where the system has not yet reached a steady state but the individual molecules sample a distribution of states along the reaction coordinate. Careful experimental design that maximizes the fraction of molecules reacting can help, but the data will always contain a mixture of states. This means that the difference electron density can be very challenging to interpret and may not provide a clear picture of the dynamics and intermediate states involved in a particular reaction. This is the major challenge of time-resolved crystallographic data analysis: how to successfully identify, interpret and model low-population states or deconvolute a mixture of states?

### 9.1. Singular value decomposition

Singular value decomposition (SVD) is one of the most established methods for deconvoluting mixtures of states and was first used in Laue time-resolved crystallographic experiments (Ren *et al.*, 2013 ▸; Schmidt *et al.*, 2003 ▸). SVD deconvolutes the data into time-independent and time-dependent vectors, ultimately allowing weighted electron-density maps to be calculated that reflect the time-independent states contributing to the measured data. Combining SVD with data collection at a range of different temperatures and/or pH values, so called five-dimensional crystallography, can be used to quantitively analyse the reaction kinetics within a crystal and guide the optimization of an experiment to access low-populated states by altering the reaction kinetics and manipulating the lifetimes of short-lived intermediates (Schmidt *et al.*, 2013 ▸).

### 9.2. Pan-data-set analysis

Pan-data-set density analysis (*PANDDA*) was originally developed to identify low-occupancy ligands in fragment-screening data sets (Pearce *et al.*, 2017 ▸). It exploits the large number of data sets typically collected during a fragment-screening campaign to make a statistical analysis of the electron-density distributions in order to identify data sets with unique features that could be low-occupancy states and ligands. Time-resolved data sets can be analysed in the same way in order to identify time-dependent changes in electron density.

### 9.3. Clustering

As discussed in Section 7.3, averaging of SSX data recorded from non-isomorphous crystals results in the loss of clear electron-density features for low-populated states. In this case, clustering of the data prior to scaling and merging can greatly improve the downstream analysis using tools such as *PANDDA*.

*Cluster*4*x* uses the correlation between the differences in C^α^ positions (or reflection amplitudes) to improve clustering prior to *PANDDA* analysis (Ginn, 2020 ▸), and we have found this to be particularly effective for the identification of weakly bound ligands in protein structures. When combined with SVD, it also aids in selecting the correct number of vectors and therefore intermediate states for time-resolved data analysis.

### 9.4. Modelling heterogeneity

Even at high resolutions, the modelling of conformational heterogeneity, or even only the modelling of multiple conformations, is often prone to subjectivity. It can therefore be beneficial to utilize large-scale computational analysis and parallelized refinement to sample large numbers of different conformation combinations in order to determine those which best describe the data. *Qfit* is a program that uses mixed integer quadratic programming (MIQP) to successfully model hidden conformations (Keedy *et al.*, 2015 ▸; Fraser *et al.*, 2011 ▸; Lang *et al.*, 2014 ▸; Riley *et al.*, 2021 ▸).

## 10. Complementary experiments

Although time-resolved crystallography can provide a huge amount of information, other biophysical and biochemical techniques are also required to obtain a full understanding of the mechanism of interest.

### 10.1. Neutron crystallography

Neutron crystallography (NMX) is a unique complement to TR-SSX experiments as it is able to experimentally determine protonation patterns. A neutron crystal structure of one or more stable or cryo-trapped intermediates can provide valuable insights, especially where the resolution of the data from TR-SSX is insufficient to reliably infer proton positions (Kono & Tamada, 2021 ▸).

### 10.2. NMR spectroscopy

Nuclear magnetic resonance (NMR) spectroscopy provides access to the thermodynamics and kinetics of protein–ligand interactions and enables a detailed picture of the equilibrium-state conditions. NMR relaxation methods can also provide insight into the populations of key states with relative populations down to ∼1% (Vallurupalli *et al.*, 2012 ▸). In addition, it can yield exchange rates and provide a wealth of other dynamic information on allosteric and conformational selection pathways (Kim *et al.*, 2017 ▸). Moreover, NMR and electron paramagnetic resonance (EPR) are perfectly suited to provide information on intrinsically disordered regions of the protein that are otherwise completely invisible using structural techniques (Drescher, 2012 ▸; Gibbs *et al.*, 2017 ▸).

### 10.3. Molecular simulations

Computational approaches such as molecular dynamics (MD) and hybrid quantum-mechanical/molecular-mechanics (QM/MM) simulations can be immensely helpful in filling in the gaps for otherwise inaccessible timescales from femto­seconds to microseconds and, with more rigorous approaches, providing energy landscapes for key chemical events along the reaction coordinate. Enhanced methods have been developed to calculate the rates of transitions between states and to include kinetic rate constants as constraints in MD simulations (Brotzakis *et al.*, 2021 ▸; Wang, Martins *et al.*, 2017 ▸). An approach that appears particularly interesting to TR-SSX experiments is to guide such simulations by experimental data (Bottaro *et al.*, 2018 ▸).

### 10.4. Electronic and vibrational spectroscopy

Spectroscopic techniques, such as UV–Vis, Raman, IR *etc.*, can provide key insights into the electronic states of metal cofactors and chromophores, radiation-damage processes and reaction kinetics and are perfectly suited to pinpointing the timings of state transitions (Balakrishnan *et al.*, 2004 ▸). Multi-dimensional spectroscopic methods can also provide additional insights into how local changes are coupled to larger-scale dynamics and are extremely complementary to time-resolved structural data.

## 11. Summary and conclusions

Time-resolved structural studies can extend our understanding of protein function by making not just structure but also function visible. With recent developments in X-ray sources, instrumentation and data-analysis tools, time-resolved experiments, which were originally the preserve of a few expert groups, are becoming simpler, can be carried out at more radiation sources and are thus increasingly accessible to a growing user base. However, these experiments are just that: discrete experiments, not just ‘data collections’. As such, careful planning and consideration of potential pitfalls is required to enable a successful experiment.

## References

[bb1] Arnlund, D., Johansson, L. C., Wickstrand, C., Barty, A., Williams, G. J., Malmerberg, E., Davidsson, J., Milathianaki, D., DePonte, D. P., Shoeman, R. L., Wang, D., James, D., Katona, G., Westenhoff, S., White, T. A., Aquila, A., Bari, S., Berntsen, P., Bogan, M., van Driel, T. B., Doak, R. B., Kjaer, K. S., Frank, M., Fromme, R., Grotjohann, I., Henning, R., Hunter, M. S., Kirian, R. A., Kosheleva, I., Kupitz, C., Liang, M., Martin, A. V., Nielsen, M. M., Messerschmidt, M., Seibert, M. M., Sjöhamn, J., Stellato, F., Weierstall, U., Zatsepin, N. A., Spence, J. C. H., Fromme, P., Schlichting, I., Boutet, S., Groenhof, G., Chapman, H. N. & Neutze, R. (2014). *Nat. Methods*, **11**, 923–926.

[bb2] Assmann, G., Brehm, W. & Diederichs, K. (2016). *J. Appl. Cryst.* **49**, 1021–1028. 10.1107/S1600576716005471 PMC4886987 27275144

[bb3] Baek, M., DiMaio, F., Anishchenko, I., Dauparas, J., Ovchinnikov, S., Lee, G. R., Wang, J., Cong, Q., Kinch, L. N., Schaeffer, R. D., Millán, C., Park, H., Adams, C., Glassman, C. R., DeGiovanni, A., Pereira, J. H., Rodrigues, A. V., van Dijk, A. A., Ebrecht, A. C., Opperman, D. J., Sagmeister, T., Buhlheller, C., Pavkov-Keller, T., Rathina­swamy, M. K., Dalwadi, U., Yip, C. K., Burke, J. E., Garcia, K. C., Grishin, N. V., Adams, P. D., Read, R. J. & Baker, D. (2021). *Science*, **373**, 871–876.

[bb4] Balakrishnan, G., Case, M. A., Pevsner, A., Zhao, X., Tengroth, C., McLendon, G. L. & Spiro, T. G. (2004). *J. Mol. Biol.* **340**, 843–856. 10.1016/j.jmb.2004.05.012 15223325

[bb5] Barends, T. R. M., Foucar, L., Ardevol, A., Nass, K., Aquila, A., Botha, S., Doak, R. B., Falahati, K., Hartmann, E., Hilpert, M., Heinz, M., Hoffmann, M. C., Köfinger, J., Koglin, J. E., Kovacsova, G., Liang, M., Milathianaki, D., Lemke, H. T., Reinstein, J., Roome, C. M., Shoeman, R. L., Williams, G. J., Burghardt, I., Hummer, G., Boutet, S. & Schlichting, I. (2015). *Science*, **350**, 445–450. 10.1126/science.aac5492 26359336

[bb6] Barends, T. R. M., Foucar, L., Botha, S., Doak, R. B., Shoeman, R. L., Nass, K., Koglin, J. E., Williams, G. J., Boutet, S., Messerschmidt, M. & Schlichting, I. (2014). *Nature*, **505**, 244–247. 10.1038/nature12773 24270807

[bb7] Bar-Even, A., Noor, E., Savir, Y., Liebermeister, W., Davidi, D., Tawfik, D. S. & Milo, R. (2011). *Biochemistry*, **50**, 4402–4410. 10.1021/bi2002289 21506553

[bb8] Barty, A., Kirian, R. A., Maia, F. R. N. C., Hantke, M., Yoon, C. H., White, T. A. & Chapman, H. (2014). *J. Appl. Cryst.* **47**, 1118–1131. 10.1107/S1600576714007626 PMC4038800 24904246

[bb9] Basu, S., Kaminski, J. W., Panepucci, E., Huang, C.-Y., Warshamanage, R., Wang, M. & Wojdyla, J. A. (2019). *J. Synchrotron Rad.* **26**, 244–252. 10.1107/S1600577518016570 PMC6337882 30655492

[bb10] Beale, J. H., Bolton, R., Marshall, S. A., Beale, E. V., Carr, S. B., Ebrahim, A., Moreno-Chicano, T., Hough, M. A., Worrall, J. A. R., Tews, I. & Owen, R. L. (2019). *J. Appl. Cryst.* **52**, 1385–1396. 10.1107/S1600576719013517 PMC6878878 31798361

[bb11] Beilsten-Edmands, J., Winter, G., Gildea, R., Parkhurst, J., Waterman, D. & Evans, G. (2020). *Acta Cryst.* D**76**, 385–399. 10.1107/S2059798320003198 PMC7137103 32254063

[bb12] Beyerlein, K. R., Dierksmeyer, D., Mariani, V., Kuhn, M., Sarrou, I., Ottaviano, A., Awel, S., Knoska, J., Fuglerud, S., Jönsson, O., Stern, S., Wiedorn, M. O., Yefanov, O., Adriano, L., Bean, R., Burkhardt, A., Fischer, P., Heymann, M., Horke, D. A., Jungnickel, K. E. J., Kovaleva, E., Lorbeer, O., Metz, M., Meyer, J., Morgan, A., Pande, K., Panneerselvam, S., Seuring, C., Tolstikova, A., Lieske, J., Aplin, S., Roessle, M., White, T. A., Chapman, H. N., Meents, A. & Oberthuer, D. (2017). *IUCrJ*, **4**, 769–777. 10.1107/S2052252517013124 PMC5668862 29123679

[bb13] Botha, S., Nass, K., Barends, T. R. M., Kabsch, W., Latz, B., Dworkowski, F., Foucar, L., Panepucci, E., Wang, M., Shoeman, R. L., Schlichting, I. & Doak, R. B. (2015). *Acta Cryst.* D**71**, 387–397. 10.1107/S1399004714026327 25664750

[bb14] Bottaro, S., Bussi, G., Kennedy, S. D., Turner, D. H. & Lindorff-Larsen, K. (2018). *Sci. Adv.* **4**, eaar8521. 10.1126/sciadv.aar8521 PMC5959319 29795785

[bb15] Boutet, S., Lomb, L., Williams, G. J., Barends, T. R. M., Aquila, A., Doak, R. B., Weierstall, U., DePonte, D. P., Steinbrener, J., Shoeman, R. L., Messerschmidt, M., Barty, A., White, T. A., Kassemeyer, S., Kirian, R. A., Seibert, M. M., Montanez, P. A., Kenney, C., Herbst, R., Hart, P., Pines, J., Haller, G., Gruner, S. M., Philipp, H. T., Tate, M. W., Hromalik, M., Koerner, L. J., van Bakel, N., Morse, J., Ghonsalves, W., Arnlund, D., Bogan, M. J., Caleman, C., Fromme, R., Hampton, C. Y., Hunter, M. S., Johansson, L. C., Katona, G., Kupitz, C., Liang, M., Martin, A. V., Nass, K., Redecke, L., Stellato, F., Timneanu, N., Wang, D., Zatsepin, N. A., Schafer, D., Defever, J., Neutze, R., Fromme, P., Spence, J. C. H., Chapman, H. N. & Schlichting, I. (2012). *Science*, **337**, 362–364.

[bb16] Bowler, M. W., Montgomery, M. G., Leslie, A. G. W. & Walker, J. E. (2006). *Acta Cryst.* D**62**, 991–995. 10.1107/S0907444906020877 16929099

[bb17] Brotzakis, Z. F., Vendruscolo, M. & Bolhuis, P. G. (2021). *Proc. Natl Acad. Sci. USA*, **118**, e2012423118. 10.1073/pnas.2012423118 PMC7812743 33376207

[bb18] Bücker, R., Hogan-Lamarre, P., Mehrabi, P., Schulz, E. C., Bultema, L. A., Gevorkov, Y., Brehm, W., Yefanov, O., Oberthür, D., Kassier, G. H. & Miller, R. J. D. (2020). *Nat. Commun.* **11**, 996. 10.1038/s41467-020-14793-0 PMC7035385 32081905

[bb19] Butryn, A., Simon, P. S., Aller, P., Hinchliffe, P., Massad, R. N., Leen, G., Tooke, C. L., Bogacz, I., Kim, I.-S., Bhowmick, A., Brewster, A. S., Devenish, N. E., Brem, J., Kamps, J. J. A. G., Lang, P. A., Rabe, P., Axford, D., Beale, J. H., Davy, B., Ebrahim, A., Orlans, J., Storm, S. L. S., Zhou, T., Owada, S., Tanaka, R., Tono, K., Evans, G., Owen, R. L., Houle, F. A., Sauter, N. K., Schofield, C. J., Spencer, J., Yachandra, V. K., Yano, J., Kern, J. F. & Orville, A. M. (2021). *Nat. Commun.* **12**, 4461. 10.1038/s41467-021-24757-7 PMC8298390 34294694

[bb20] Chang, A., Jeske, L., Ulbrich, S., Hofmann, J., Koblitz, J., Schomburg, I., Neumann-Schaal, M., Jahn, D. & Schomburg, D. (2021). *Nucleic Acids Res.* **49**, D498–D508. 10.1093/nar/gkaa1025 PMC7779020 33211880

[bb21] Chapman, H. N. (2019). *Annu. Rev. Biochem.* **88**, 35–58. 10.1146/annurev-biochem-013118-110744 30601681

[bb22] Chapman, H. N., Fromme, P., Barty, A., White, T., Kirian, R. A., Aquila, A., Hunter, M. S., Schulz, J., DePonte, D. P., Weierstall, U., Doak, R. B., Maia, F. R. N. C., Martin, A. V., Schlichting, I., Lomb, L., Coppola, N., Shoeman, R. L., Epp, S. W., Hartmann, R., Rolles, D., Rudenko, A., Foucar, L., Kimmel, N., Weidenspointner, G., Holl, P., Liang, M., Barthelmess, M., Caleman, C., Boutet, S., Bogan, M. J., Krzywinski, J., Bostedt, C., Bajt, S., Gumprecht, L., Rudek, B., Erk, B., Schmidt, C., Hömke, A., Reich, C., Pietschner, D., Strüder, L., Hauser, G., Gorke, H., Ullrich, J., Herrmann, S., Schaller, G., Schopper, F., Soltau, H., Kühnel, K., Messer­schmidt, M., Bozek, J. D., Hau-Riege, S. P., Frank, M., Hampton, C. Y., Sierra, R. G., Starodub, D., Williams, G. J., Hajdu, J., Timneanu, N., Seibert, M. M., Andreasson, J., Rocker, A., Jönsson, O., Svenda, M., Stern, S., Nass, K., Andritschke, R., Schröter, C., Krasniqi, F., Bott, M., Schmidt, K. E., Wang, X., Grotjohann, I., Holton, J. M., Barends, T. R. M., Neutze, R., Marchesini, S., Fromme, R., Schorb, S., Rupp, D., Adolph, M., Gorkhover, T., Andersson, I., Hirsemann, H., Potdevin, G., Graafsma, H., Nilsson, B. & Spence, J. C. H. (2011). *Nature*, **470**, 73–77.

[bb23] Cheng, R. (2020). *Crystals*, **10**, 215.

[bb24] Coquelle, N., Sliwa, M., Woodhouse, J., Schirò, G., Adam, V., Aquila, A., Barends, T. R. M., Boutet, S., Byrdin, M., Carbajo, S., De la Mora, E., Doak, R. B., Feliks, M., Fieschi, F., Foucar, L., Guillon, V., Hilpert, M., Hunter, M. S., Jakobs, S., Koglin, J. E., Kovacsova, G., Lane, T. J., Lévy, B., Liang, M., Nass, K., Ridard, J., Robinson, J. S., Roome, C. M., Ruckebusch, C., Seaberg, M., Thepaut, M., Cammarata, M., Demachy, I., Field, M., Shoeman, R. L., Bourgeois, D., Colletier, J.-P., Schlichting, I. & Weik, M. (2018). *Nat. Chem.* **10**, 31–37. 10.1038/nchem.2853 29256511

[bb25] Dauter, Z. (1999). *Acta Cryst.* D**55**, 1703–1717. 10.1107/s0907444999008367 10531520

[bb26] Davy, B., Axford, D., Beale, J. H., Butryn, A., Docker, P., Ebrahim, A., Leen, G., Orville, A. M., Owen, R. L. & Aller, P. (2019). *J. Synchrotron Rad.* **26**, 1820–1825. 10.1107/S1600577519009329 PMC6730619 31490175

[bb27] Drescher, M. (2012). *Top. Curr. Chem.* **321**, 91–119. 10.1007/128_2011_235 21826602

[bb28] Ebrahim, A., Moreno-Chicano, T., Appleby, M. V., Chaplin, A. K., Beale, J. H., Sherrell, D. A., Duyvesteyn, H. M. E., Owada, S., Tono, K., Sugimoto, H., Strange, R. W., Worrall, J. A. R., Axford, D., Owen, R. L. & Hough, M. A. (2019). *IUCrJ*, **6**, 543–551. 10.1107/S2052252519003956 PMC6608622 31316799

[bb29] Evans, P. R. & Murshudov, G. N. (2013). *Acta Cryst.* D**69**, 1204–1214. 10.1107/S0907444913000061 PMC3689523 23793146

[bb30] Foadi, J., Aller, P., Alguel, Y., Cameron, A., Axford, D., Owen, R. L., Armour, W., Waterman, D. G., Iwata, S. & Evans, G. (2013). *Acta Cryst.* D**69**, 1617–1632. 10.1107/S0907444913012274 PMC3727331 23897484

[bb31] Fraser, J. S., van den Bedem, H., Samelson, A. J., Lang, P. T., Holton, J. M., Echols, N. & Alber, T. (2011). *Proc. Natl Acad. Sci. USA*, **108**, 16247–16252. 10.1073/pnas.1111325108 PMC3182744 21918110

[bb33] Fuller, F. D., Gul, S., Chatterjee, R., Burgie, E. S., Young, I. D., Lebrette, H., Srinivas, V., Brewster, A. S., Michels-Clark, T., Clinger, J. A., Andi, B., Ibrahim, M., Pastor, E., de Lichtenberg, C., Hussein, R., Pollock, C. J., Zhang, M., Stan, C. A., Kroll, T., Fransson, T., Weninger, C., Kubin, M., Aller, P., Lassalle, L., Bräuer, P., Miller, M. D., Amin, M., Koroidov, S., Roessler, C. G., Allaire, M., Sierra, R. G., Docker, P. T., Glownia, J. M., Nelson, S., Koglin, J. E., Zhu, D., Chollet, M., Song, S., Lemke, H., Liang, M., Sokaras, D., Alonso-Mori, R., Zouni, A., Messinger, J., Bergmann, U., Boal, A. K., Bollinger, J. M., Krebs, C., Högbom, M., Phillips, G. N., Vierstra, R. D., Sauter, N. K., Orville, A. M., Kern, J., Yachandra, V. K. & Yano, J. (2017). *Nat. Methods*, **14**, 443–449. 10.1038/nmeth.4195 PMC5376230 28250468

[bb34] Gati, C., Bourenkov, G., Klinge, M., Rehders, D., Stellato, F., Oberthür, D., Yefanov, O., Sommer, B. P., Mogk, S., Duszenko, M., Betzel, C., Schneider, T. R., Chapman, H. N. & Redecke, L. (2014). *IUCrJ*, **1**, 87–94. 10.1107/S2052252513033939 PMC4062088 25075324

[bb35] Gibbs, E. B., Cook, E. C. & Showalter, S. A. (2017). *Arch. Biochem. Biophys.* **628**, 57–70. 10.1016/j.abb.2017.05.008 28502465

[bb36] Ginn, H. M. (2020). *Acta Cryst.* D**76**, 1134–1144. 10.1107/S2059798320012619 PMC7604910 33135684

[bb37] Giordano, R., Leal, R. M. F., Bourenkov, G. P., McSweeney, S. & Popov, A. N. (2012). *Acta Cryst.* D**68**, 649–658. 10.1107/S0907444912006841 22683787

[bb38] Gorel, A., Schlichting, I. & Barends, T. R. M. (2021). *IUCrJ*, **8**, 532–543. 10.1107/S205225252100467X PMC8256713 34258002

[bb39] Gotthard, G., Aumonier, S., De Sanctis, D., Leonard, G., von Stetten, D. & Royant, A. (2019). *IUCrJ*, **6**, 665–680. 10.1107/S205225251900616X PMC6608634 31316810

[bb40] Grünbein, M. L. & Nass Kovacs, G. (2019). *Acta Cryst.* D**75**, 178–191. 10.1107/S205979831801567X PMC6400261 30821706

[bb41] Grünbein, M. L., Stricker, M., Nass Kovacs, G., Kloos, M., Doak, R. B., Shoeman, R. L., Reinstein, J., Lecler, S., Haacke, S. & Schlichting, I. (2020). *Nat. Methods*, **17**, 681–684. 10.1038/s41592-020-0847-3 32451477

[bb42] Harata, K. & Akiba, T. (2006). *Acta Cryst.* D**62**, 375–382. 10.1107/S0907444906001314 16552138

[bb43] Karplus, P. A. & Diederichs, K. (2012). *Science*, **336**, 1030–1033. 10.1126/science.1218231 PMC3457925 22628654

[bb44] Karplus, P. A. & Diederichs, K. (2015). *Curr. Opin. Struct. Biol.* **34**, 60–68. 10.1016/j.sbi.2015.07.003 PMC4684713 26209821

[bb45] Ke, T.-W., Brewster, A. S., Yu, S. X., Ushizima, D., Yang, C. & Sauter, N. K. (2018). *J. Synchrotron Rad.* **25**, 655–670. 10.1107/S1600577518004873 PMC5929353 29714177

[bb46] Keedy, D. A., Fraser, J. S. & van den Bedem, H. (2015). *PLoS Comput. Biol.* **11**, e1004507. 10.1371/journal.pcbi.1004507 PMC4624436 26506617

[bb47] Kern, J., Alonso-Mori, R., Tran, R., Hattne, J., Gildea, R. J., Echols, N., Glöckner, C., Hellmich, J., Laksmono, H., Sierra, R. G., Lassalle-Kaiser, B., Koroidov, S., Lampe, A., Han, G., Gul, S., Difiore, D., Milathianaki, D., Fry, A. R., Miahnahri, A., Schafer, D. W., Messerschmidt, M., Seibert, M. M., Koglin, J. E., Sokaras, D., Weng, T.-C., Sellberg, J., Latimer, M. J., Grosse-Kunstleve, R. W., Zwart, P. H., White, W. E., Glatzel, P., Adams, P. D., Bogan, M. J., Williams, G. J., Boutet, S., Messinger, J., Zouni, A., Sauter, N. K., Yachandra, V. K., Bergmann, U. & Yano, J. (2013). *Science*, **340**, 491–495.

[bb48] Kesgin–Schaefer, S., Heidemann, J., Puchert, A., Koelbel, K., Yorke, B. A., Huse, N., Pearson, A. R., Uetrecht, C. & Tidow, H. (2019). *FEBS J.* **286**, 2329–2340. 10.1111/febs.14797 30817081

[bb49] Kim, T. H., Mehrabi, P., Ren, Z., Sljoka, A., Ing, C., Bezginov, A., Ye, L., Pomès, R., Prosser, R. S. & Pai, E. F. (2017). *Science*, **355**, eaag2355. 10.1126/science.aag2355 28104837

[bb50] Kono, F. & Tamada, T. (2021). *Curr. Opin. Struct. Biol.* **71**, 36–42. 10.1016/j.sbi.2021.05.007 34214927

[bb51] Konold, P. E., Arik, E., Weissenborn, J., Arents, J. C., Hellingwerf, K. J., van Stokkum, I. H. M., Kennis, J. T. M. & Groot, M. L. (2020). *Nat. Commun.* **11**, 4248. 10.1038/s41467-020-18065-9 PMC7447820 32843623

[bb52] Kupitz, C., Basu, S., Grotjohann, I., Fromme, R., Zatsepin, N. A., Rendek, K. N., Hunter, M. S., Shoeman, R. L., White, T. A., Wang, D., James, D., Yang, J.-H., Cobb, D. E., Reeder, B., Sierra, R. G., Liu, H., Barty, A., Aquila, A. L., Deponte, D., Kirian, R. A., Bari, S., Bergkamp, J. J., Beyerlein, K. R., Bogan, M. J., Caleman, C., Chao, T.-C., Conrad, C. E., Davis, K. M., Fleckenstein, H., Galli, L., Hau-Riege, S. P., Kassemeyer, S., Laksmono, H., Liang, M., Lomb, L., Marchesini, S., Martin, A. V., Messerschmidt, M., Milathianaki, D., Nass, K., Ros, A., Roy-Chowdhury, S., Schmidt, K., Seibert, M., Steinbrener, J., Stellato, F., Yan, L., Yoon, C., Moore, T. A., Moore, A. L., Pushkar, Y., Williams, G. J., Boutet, S., Doak, R. B., Weierstall, U., Frank, M., Chapman, H. N., Spence, J. C. H. & Fromme, P. (2014). *Nature*, **513**, 261–265.

[bb53] Lang, P. T., Holton, J. M., Fraser, J. S. & Alber, T. (2014). *Proc. Natl Acad. Sci. USA*, **111**, 237–242.

[bb54] Levantino, M., Yorke, B. A., Monteiro, D. C. F., Cammarata, M. & Pearson, A. R. (2015). *Curr. Opin. Struct. Biol.* **35**, 41–48. 10.1016/j.sbi.2015.07.017 26342489

[bb55] Lieske, J., Cerv, M., Kreida, S., Komadina, D., Fischer, J., Barthelmess, M., Fischer, P., Pakendorf, T., Yefanov, O., Mariani, V., Seine, T., Ross, B. H., Crosas, E., Lorbeer, O., Burkhardt, A., Lane, T. J., Guenther, S., Bergtholdt, J., Schoen, S., Törnroth-Horsefield, S., Chapman, H. N. & Meents, A. (2019). *IUCrJ*, **6**, 714–728. 10.1107/S2052252519007395 PMC6608620 31316815

[bb56] Makinen, M. W. & Fink, A. L. (1977). *Annu. Rev. Biophys. Bioeng.* **6**, 301–343. 10.1146/annurev.bb.06.060177.001505 194529

[bb57] Mariani, V., Morgan, A., Yoon, C. H., Lane, T. J., White, T. A., O’Grady, C., Kuhn, M., Aplin, S., Koglin, J., Barty, A. & Chapman, H. N. (2016). *J. Appl. Cryst.* **49**, 1073–1080. 10.1107/S1600576716007469 PMC4886993 27275150

[bb58] Martiel, I., Müller-Werkmeister, H. M. & Cohen, A. E. (2019). *Acta Cryst.* D**75**, 160–177. 10.1107/S2059798318017953 PMC6400256 30821705

[bb59] Martin, R. W. & Zilm, K. W. (2003). *J. Magn. Reson.* **165**, 162–174. 10.1016/s1090-7807(03)00253-2 14568526

[bb60] Mehrabi, P., Bücker, R., Bourenkov, G., Ginn, H. M., von Stetten, D., Müller-Werkmeister, H. M., Kuo, A., Morizumi, T., Eger, B. T., Ou, W.-L., Oghbaey, S., Sarracini, A., Besaw, J. E., Paré-Labrosse, O., Meier, S., Schikora, H., Tellkamp, F., Marx, A., Sherrell, D. A., Axford, D., Owen, R. L., Ernst, O. P., Pai, E. F., Schulz, E. C. & Miller, R. J. D. (2021). *Sci. Adv.* **7**, eabf1380. 10.1126/sciadv.abf1380 PMC7968842 33731353

[bb61] Mehrabi, P., Müller-Werkmeister, H. M., Leimkohl, J.-P., Schikora, H., Ninkovic, J., Krivokuca, S., Andriček, L., Epp, S. W., Sherrell, D., Owen, R. L., Pearson, A. R., Tellkamp, F., Schulz, E. C. & Miller, R. J. D. (2020). *J. Synchrotron Rad.* **27**, 360–370. 10.1107/S1600577520000685 PMC7064102 32153274

[bb62] Mehrabi, P., Schulz, E. C., Agthe, M., Horrell, S., Bourenkov, G., von Stetten, D., Leimkohl, J.-P., Schikora, H., Schneider, T. R., Pearson, A. R., Tellkamp, F. & Miller, R. J. D. (2019). *Nat. Methods*, **16**, 979–982. 10.1038/s41592-019-0553-1 31527838

[bb63] Mehrabi, P., Schulz, E. C., Dsouza, R., Müller-Werkmeister, H. M., Tellkamp, F., Miller, R. J. D. & Pai, E. F. (2019). *Science*, **365**, 1167–1170. 10.1126/science.aaw9904 31515393

[bb64] Moffat, K. (2001). *Chem. Rev.* **101**, 1569–1582. 10.1021/cr990039q 11709992

[bb65] Monteiro, D. C. F., Amoah, E., Rogers, C. & Pearson, A. R. (2021). *Acta Cryst.* D**77**, 1218–1232. 10.1107/S2059798321008809 PMC8489231 34605426

[bb66] Monteiro, D. C. F., von Stetten, D., Stohrer, C., Sans, M., Pearson, A. R., Santoni, G., van der Linden, P. & Trebbin, M. (2020). *IUCrJ*, **7**, 207–219. 10.1107/S2052252519016865 PMC7055382 32148849

[bb67] Mora, E. de la, Coquelle, N., Bury, C. S., Rosenthal, M., Holton, J. M., Carmichael, I., Garman, E. F., Burghammer, M., Colletier, J.-P. & Weik, M. (2020). *Proc. Natl Acad. Sci.* **117**, 4142–4151. 10.1073/pnas.1821522117 PMC7049125 32047034

[bb68] Moreno-Chicano, T., Ebrahim, A., Axford, D., Appleby, M. V., Beale, J. H., Chaplin, A. K., Duyvesteyn, H. M. E., Ghiladi, R. A., Owada, S., Sherrell, D. A., Strange, R. W., Sugimoto, H., Tono, K., Worrall, J. A. R., Owen, R. L. & Hough, M. A. (2019). *IUCrJ*, **6**, 1074–1085. 10.1107/S2052252519011655 PMC6830213 31709063

[bb69] Murray, T. D., Lyubimov, A. Y., Ogata, C. M., Vo, H., Uervirojnangkoorn, M., Brunger, A. T. & Berger, J. M. (2015). *Acta Cryst.* D**71**, 1987–1997. 10.1107/S1399004715015011 PMC4601365 26457423

[bb70] Nango, E., Royant, A., Kubo, M., Nakane, T., Wickstrand, C., Kimura, T., Tanaka, T., Tono, K., Song, C., Tanaka, R., Arima, T., Yamashita, A., Kobayashi, J., Hosaka, T., Mizohata, E., Nogly, P., Sugahara, M., Nam, D., Nomura, T., Shimamura, T., Im, D., Fujiwara, T., Yamanaka, Y., Jeon, B., Nishizawa, T., Oda, K., Fukuda, M., Andersson, R., Båth, P., Dods, R., Davidsson, J., Matsuoka, S., Kawatake, S., Murata, M., Nureki, O., Owada, S., Kameshima, T., Hatsui, T., Joti, Y., Schertler, G., Yabashi, M., Bondar, A.-N., Standfuss, J., Neutze, R. & Iwata, S. (2016). *Science*, **354**, 1552–1557. 10.1126/science.aah3497 28008064

[bb71] Nass, K., Gorel, A., Abdullah, M. M. V., Martin, A., Kloos, M., Marinelli, A., Aquila, A., Barends, T. R. M., Decker, F.-J., Doak, R. B., Foucar, L., Hartmann, E., Hilpert, M., Hunter, M. S., Jurek, Z., Koglin, J. E., Kozlov, A., Lutman, A. A., Kovacs, G. N., Roome, C. M., Shoeman, R. L., Santra, R., Quiney, H. M., Ziaja, B., Boutet, S. & Schlichting, I. (2020). *Nat. Commun.* **11**, 1814. 10.1038/s41467-020-15610-4 PMC7156470 32286284

[bb72] Nogly, P., James, D., Wang, D., White, T. A., Zatsepin, N., Shilova, A., Nelson, G., Liu, H., Johansson, L., Heymann, M., Jaeger, K., Metz, M., Wickstrand, C., Wu, W., Båth, P., Berntsen, P., Oberthuer, D., Panneels, V., Cherezov, V., Chapman, H., Schertler, G., Neutze, R., Spence, J., Moraes, I., Burghammer, M., Standfuss, J. & Weierstall, U. (2015). *IUCrJ*, **2**, 168–176. 10.1107/S2052252514026487 PMC4392771 25866654

[bb73] Nogly, P., Weinert, T., James, D., Carbajo, S., Ozerov, D., Furrer, A., Gashi, D., Borin, V., Skopintsev, P., Jaeger, K., Nass, K., Båth, P., Bosman, R., Koglin, J., Seaberg, M., Lane, T., Kekilli, D., Brünle, S., Tanaka, T., Wu, W., Milne, C., White, T., Barty, A., Weierstall, U., Panneels, V., Nango, E., Iwata, S., Hunter, M., Schapiro, I., Schertler, G., Neutze, R. & Standfuss, J. (2018). *Science*, **361**, eaat0094. 10.1126/science.aat0094 29903883

[bb74] Norton-Baker, B., Mehrabi, P., Boger, J., Schönherr, R., von Stetten, D., Schikora, H., Kwok, A. O., Martin, R. W., Miller, R. J. D., Redecke, L. & Schulz, E. C. (2021). *Acta Cryst.* D**77**, 820–834. 10.1107/S2059798321003855 PMC8171066 34076595

[bb75] Oghbaey, S., Sarracini, A., Ginn, H. M., Pare-Labrosse, O., Kuo, A., Marx, A., Epp, S. W., Sherrell, D. A., Eger, B. T., Zhong, Y., Loch, R., Mariani, V., Alonso-Mori, R., Nelson, S., Lemke, H. T., Owen, R. L., Pearson, A. R., Stuart, D. I., Ernst, O. P., Mueller-Werkmeister, H. M. & Miller, R. J. D. (2016). *Acta Cryst.* D**72**, 944–955. 10.1107/S2059798316010834 PMC5937680 27487825

[bb76] Orville, A. M. (2020). *Curr. Opin. Struct. Biol.* **65**, 193–208. 10.1016/j.sbi.2020.08.011 33049498

[bb77] Pande, K., Hutchison, C. D. M., Groenhof, G., Aquila, A., Robinson, J. S., Tenboer, J., Basu, S., Boutet, S., DePonte, D. P., Liang, M., White, T. A., Zatsepin, N. A., Yefanov, O., Morozov, D., Oberthuer, D., Gati, C., Subramanian, G., James, D., Zhao, Y., Koralek, J., Brayshaw, J., Kupitz, C., Conrad, C., Roy-Chowdhury, S., Coe, J. D., Metz, M., Xavier, P. L., Grant, T. D., Koglin, J. E., Ketawala, G., Fromme, R., Šrajer, V., Henning, R., Spence, J. C. H., Ourmazd, A., Schwander, P., Weierstall, U., Frank, M., Fromme, P., Barty, A., Chapman, H. N., Moffat, K., van Thor, J. J. & Schmidt, M. (2016). *Science*, **352**, 725–729.

[bb78] Pearce, N. M., Krojer, T., Bradley, A. R., Collins, P., Nowak, R. P., Talon, R., Marsden, B. D., Kelm, S., Shi, J., Deane, C. M. & von Delft, F. (2017). *Nat. Commun.* **8**, 15123. 10.1038/ncomms15123 PMC5413968 28436492

[bb79] Pearson, A. R. & Mehrabi, P. (2020). *Curr. Opin. Struct. Biol.* **65**, 168–174. 10.1016/j.sbi.2020.06.019 32846363

[bb80] Pearson, A. R. & Mozzarelli, A. (2011). *Biochim. Biophys. Acta*, **1814**, 731–733. 10.1016/j.bbapap.2011.04.010 21555112

[bb81] Pearson, A. R., Mozzarelli, A. & Rossi, G. L. (2004). *Curr. Opin. Struct. Biol.* **14**, 656–662. 10.1016/j.sbi.2004.10.007 15582388

[bb82] Ramakrishnan, S., Stagno, J. R., Conrad, C. E., Ding, J., Yu, P., Bhandari, Y. R., Lee, Y.-T., Pauly, G., Yefanov, O., Wiedorn, M. O., Knoska, J., Oberthür, D., White, T. A., Barty, A., Mariani, V., Li, C., Brehm, W., Heinz, W. F., Magidson, V., Lockett, S., Hunter, M. S., Boutet, S., Zatsepin, N. A., Zuo, X., Grant, T. D., Pandey, S., Schmidt, M., Spence, J. C. H., Chapman, H. N. & Wang, Y.-X. (2021). *Nat. Commun.* **12**, 1762. 10.1038/s41467-021-21838-5 PMC7979858 33741910

[bb83] Redecke, L., Nass, K., DePonte, D. P., White, T. A., Rehders, D., Barty, A., Stellato, F., Liang, M., Barends, T. R. M., Boutet, S., Williams, G. J., Messerschmidt, M., Seibert, M. M., Aquila, A., Arnlund, D., Bajt, S., Barth, T., Bogan, M. J., Caleman, C., Chao, T.-C., Doak, R. B., Fleckenstein, H., Frank, M., Fromme, R., Galli, L., Grotjohann, I., Hunter, M. S., Johansson, L. C., Kassemeyer, S., Katona, G., Kirian, R. A., Koopmann, R., Kupitz, C., Lomb, L., Martin, A. V., Mogk, S., Neutze, R., Shoeman, R. L., Steinbrener, J., Timneanu, N., Wang, D., Weierstall, U., Zatsepin, N. A., Spence, J. C. H., Fromme, P., Schlichting, I., Duszenko, M., Betzel, C. & Chapman, H. N. (2013). *Science*, **339**, 227–230.

[bb84] Ren, Z., Bourgeois, D., Helliwell, J. R., Moffat, K., Šrajer, V. & Stoddard, B. L. (1999). *J. Synchrotron Rad.* **6**, 891–917.

[bb85] Ren, Z., Chan, P. W. Y., Moffat, K., Pai, E. F., Royer, W. E., Šrajer, V. & Yang, X. (2013). *Acta Cryst.* D**69**, 946–959. 10.1107/S0907444913003454 PMC3663119 23695239

[bb86] Ren, Z. & Moffat, K. (1994). *J. Synchrotron Rad.* **1**, 78–82. 10.1107/S0909049594006692 16728788

[bb87] Richter, M. (2013). *Nat. Prod. Rep.* **30**, 1324. 10.1039/c3np70045c 23934236

[bb88] Riley, B. T., Wankowicz, S. A., de Oliveira, S. H. P., van Zundert, G. C. P., Hogan, D. W., Fraser, J. S., Keedy, D. A. & van den Bedem, H. (2021). *Protein Sci.* **30**, 270–285. 10.1002/pro.4001 PMC7737783 33210433

[bb89] Roedig, P., Duman, R., Sanchez-Weatherby, J., Vartiainen, I., Burkhardt, A., Warmer, M., David, C., Wagner, A. & Meents, A. (2016). *J. Appl. Cryst.* **49**, 968–975. 10.1107/S1600576716006348 PMC4886986 27275143

[bb90] Roedig, P., Ginn, H. M., Pakendorf, T., Sutton, G., Harlos, K., Walter, T. S., Meyer, J., Fischer, P., Duman, R., Vartiainen, I., Reime, B., Warmer, M., Brewster, A. S., Young, I. D., Michels-Clark, T., Sauter, N. K., Kotecha, A., Kelly, J., Rowlands, D. J., Sikorsky, M., Nelson, S., Damiani, D. S., Alonso-Mori, R., Ren, J., Fry, E. E., David, C., Stuart, D. I., Wagner, A. & Meents, A. (2017). *Nat. Methods*, **14**, 805–810. 10.1038/nmeth.4335 PMC5588887 28628129

[bb91] Roessler, C. G., Kuczewski, A., Stearns, R., Ellson, R., Olechno, J., Orville, A. M., Allaire, M., Soares, A. S. & Héroux, A. (2013). *J. Synchrotron Rad.* **20**, 805–808. 10.1107/S0909049513020372 PMC3747951 23955046

[bb92] Russo Krauss, I., Sica, F., Mattia, C. A. & Merlino, A. (2012). *Int. J. Mol. Sci.* **13**, 3782–3800. 10.3390/ijms13033782 PMC3317743 22489183

[bb93] Sanchez-Weatherby, J., Bowler, M. W., Huet, J., Gobbo, A., Felisaz, F., Lavault, B., Moya, R., Kadlec, J., Ravelli, R. B. G. & Cipriani, F. (2009). *Acta Cryst.* D**65**, 1237–1246. 10.1107/S0907444909037822 19966409

[bb94] Santoni, G., Zander, U., Mueller-Dieckmann, C., Leonard, G. & Popov, A. (2017). *J. Appl. Cryst.* **50**, 1844–1851. 10.1107/S1600576717015229 PMC5713145 29217993

[bb95] Schlichting, I. (2015). *IUCrJ*, **2**, 246–255. 10.1107/S205225251402702X PMC4392417 25866661

[bb96] Schmidt, M. (2013). *Adv. Condens. Matter Phys.* **2013**, 1–10.

[bb97] Schmidt, M. (2020). *Crystals*, **10**, 116.

[bb98] Schmidt, M., Rajagopal, S., Ren, Z. & Moffat, K. (2003). *Biophys. J.* **84**, 2112–2129. 10.1016/S0006-3495(03)75018-8 PMC1302779 12609912

[bb99] Schmidt, M., Srajer, V., Henning, R., Ihee, H., Purwar, N., Tenboer, J. & Tripathi, S. (2013). *Acta Cryst.* D**69**, 2534–2542. 10.1107/S0907444913025997 PMC3852658 24311594

[bb100] Schulz, E. C., Mehrabi, P., Müller-Werkmeister, H. M., Tellkamp, F., Jha, A., Stuart, W., Persch, E., De Gasparo, R., Diederich, F., Pai, E. F. & Miller, R. J. D. (2018). *Nat. Methods*, **15**, 901–904. 10.1038/s41592-018-0180-2 30377366

[bb101] Sherrell, D. A., Foster, A. J., Hudson, L., Nutter, B., O’Hea, J., Nelson, S., Paré-Labrosse, O., Oghbaey, S., Miller, R. J. D. & Owen, R. L. (2015). *J. Synchrotron Rad.* **22**, 1372–1378. 10.1107/S1600577515016938 PMC4629865 26524301

[bb102] Specht, A., Ursby, T., Weik, M., Peng, L., Kroon, J., Bourgeois, D. & Goeldner, M. (2001). *ChemBioChem*, **2**, 845. 10.1002/1439-7633(20011105)2:11<845::AID-CBIC845>3.0.CO;2-C 11948871

[bb103] Stagno, J. R., Liu, Y., Bhandari, Y. R., Conrad, C. E., Panja, S., Swain, M., Fan, L., Nelson, G., Li, C., Wendel, D. R., White, T. A., Coe, J. D., Wiedorn, M. O., Knoska, J., Oberthuer, D., Tuckey, R. A., Yu, P., Dyba, M., Tarasov, S. G., Weierstall, U., Grant, T. D., Schwieters, C. D., Zhang, J., Ferré-D’Amaré, A. R., Fromme, P., Draper, D. E., Liang, M., Hunter, M. S., Boutet, S., Tan, K., Zuo, X., Ji, X., Barty, A., Zatsepin, N. A., Chapman, H. N., Spence, J. C. H., Woodson, S. A. & Wang, Y.-X. (2017). *Nature*, **541**, 242–246. 10.1038/nature20599 PMC5502819 27841871

[bb104] Stellato, F., Oberthür, D., Liang, M., Bean, R., Gati, C., Yefanov, O., Barty, A., Burkhardt, A., Fischer, P., Galli, L., Kirian, R. A., Meyer, J., Panneerselvam, S., Yoon, C. H., Chervinskii, F., Speller, E., White, T. A., Betzel, C., Meents, A. & Chapman, H. N. (2014). *IUCrJ*, **1**, 204–212. 10.1107/S2052252514010070 PMC4107920 25075341

[bb105] Stohrer, C., Horrell, S., Meier, S., Sans, M., von Stetten, D., Hough, M., Goldman, A., Monteiro, D. C. F. & Pearson, A. R. (2021). *Acta Cryst.* D**77**, 194–204. 10.1107/S2059798320015454 PMC7869895 33559608

[bb106] Tenboer, J., Basu, S., Zatsepin, N., Pande, K., Milathianaki, D., Frank, M., Hunter, M., Boutet, S., Williams, G. J., Koglin, J. E., Oberthuer, D., Heymann, M., Kupitz, C., Conrad, C., Coe, J., Roy-Chowdhury, S., Weierstall, U., James, D., Wang, D., Grant, T., Barty, A., Yefanov, O., Scales, J., Gati, C., Seuring, C., Srajer, V., Henning, R., Schwander, P., Fromme, R., Ourmazd, A., Moffat, K., Van Thor, J. J., Spence, J. C. H., Fromme, P., Chapman, H. N. & Schmidt, M. (2014). *Science*, **346**, 1242–1246. 10.1126/science.1259357 PMC4361027 25477465

[bb107] Tunyasuvunakool, K., Adler, J., Wu, Z., Green, T., Zielinski, M., Žídek, A., Bridgland, A., Cowie, A., Meyer, C., Laydon, A., Velankar, S., Kleywegt, G. J., Bateman, A., Evans, R., Pritzel, A., Figurnov, M., Ronneberger, O., Bates, R., Kohl, S. A. A., Potapenko, A., Ballard, A. J., Romera-Paredes, B., Nikolov, S., Jain, R., Clancy, E., Reiman, D., Petersen, S., Senior, A. W., Kavukcuoglu, K., Birney, E., Kohli, P., Jumper, J. & Hassabis, D. (2021). *Nature*, **596**, 590–596.

[bb108] Vallurupalli, P., Bouvignies, G. & Kay, L. E. (2012). *J. Am. Chem. Soc.* **134**, 8148–8161. 10.1021/ja3001419 22554188

[bb109] Wang, J., Brudvig, G. W., Batista, V. S. & Moore, P. B. (2017). *Protein Sci.* **26**, 2410–2416. 10.1002/pro.3314 PMC5699489 28960580

[bb110] Wang, Y., Martins, J. M. & Lindorff-Larsen, K. (2017). *Chem. Sci.* **8**, 6466–6473. 10.1039/c7sc01627a PMC5859887 29619200

[bb111] Weinert, T., Olieric, N., Cheng, R., Brünle, S., James, D., Ozerov, D., Gashi, D., Vera, L., Marsh, M., Jaeger, K., Dworkowski, F., Panepucci, E., Basu, S., Skopintsev, P., Doré, A. S., Geng, T., Cooke, R. M., Liang, M., Prota, A. E., Panneels, V., Nogly, P., Ermler, U., Schertler, G., Hennig, M., Steinmetz, M. O., Wang, M. & Standfuss, J. (2017). *Nat. Commun.* **8**, 542. 10.1038/s41467-017-00630-4 PMC5599499 28912485

[bb112] Weinert, T., Skopintsev, P., James, D., Dworkowski, F., Panepucci, E., Kekilli, D., Furrer, A., Brünle, S., Mous, S., Ozerov, D., Nogly, P., Wang, M. & Standfuss, J. (2019). *Science*, **365**, 61–65. 10.1126/science.aaw8634 31273117

[bb113] White, T. A., Kirian, R. A., Martin, A. V., Aquila, A., Nass, K., Barty, A. & Chapman, H. N. (2012). *J. Appl. Cryst.* **45**, 335–341.

[bb114] White, T. A., Mariani, V., Brehm, W., Yefanov, O., Barty, A., Beyerlein, K. R., Chervinskii, F., Galli, L., Gati, C., Nakane, T., Tolstikova, A., Yamashita, K., Yoon, C. H., Diederichs, K. & Chapman, H. N. (2016). *J. Appl. Cryst.* **49**, 680–689. 10.1107/S1600576716004751 PMC4815879 27047311

[bb115] Wilmot, C. M., Sjögren, T., Carlsson, G. H., Berglund, G. I. & Hajdu, J. (2002). *Methods Enzymol.* **353**, 301–318. 10.1016/s0076-6879(02)53057-3 12078505

[bb116] Yorke, B. A., Beddard, G. S., Owen, R. L. & Pearson, A. R. (2014). *Nat. Methods*, **11**, 1131–1134. 10.1038/nmeth.3139 PMC4216935 25282611

[bb117] Zander, U., Bourenkov, G., Popov, A. N., de Sanctis, D., Svensson, O., McCarthy, A. A., Round, E., Gordeliy, V., Mueller-Dieckmann, C. & Leonard, G. A. (2015). *Acta Cryst.* D**71**, 2328–2343. 10.1107/S1399004715017927 PMC4631482 26527148

[bb118] Zander, U., Cianci, M., Foos, N., Silva, C. S., Mazzei, L., Zubieta, C., de Maria, A. & Nanao, M. H. (2016). *Acta Cryst.* D**72**, 1026–1035. 10.1107/S2059798316012079 PMC5013596 27599735

[bb119] Zarrine-Afsar, A., Barends, T. R. M., Müller, C., Fuchs, M. R., Lomb, L., Schlichting, I. & Miller, R. J. D. (2012). *Acta Cryst.* D**68**, 321–323. 10.1107/S0907444911055296 22349234

[bb120] Zeldin, O. B., Gerstel, M. & Garman, E. F. (2013). *J. Appl. Cryst.* **46**, 1225–1230.

[bb121] Zhao, F.-Z., Sun, B., Yu, L., Xiao, Q.-J., Wang, Z.-J., Chen, L.-L., Liang, H., Wang, Q.-S., He, J.-H. & Yin, D.-C. (2020). *Lab Chip*, **20**, 3888–3898. 10.1039/d0lc00443j 32966481

